# Muscle oxidative metabolism accelerates with mild acidosis during incremental intermittent isometric plantar flexion exercise

**DOI:** 10.1186/1476-5918-4-2

**Published:** 2005-02-20

**Authors:** Toshiyuki Homma, Takafumi Hamaoka, Takayuki Sako, Motohide Murakami, Kazuki Esaki, Ryotaro Kime, Toshihito Katsumura

**Affiliations:** 1 Department of Preventive Medicine and Public Health, Tokyo Medical University, 6-1-1 Shinjuku, Shinjuku-ku, Tokyo, 160-8402, Japan; 2 Department of Sports Sciences, Japan Institute of Sports Sciences, 3-15-1 Nishigaoka, Kita-ku, Tokyo, 115-0056, Japan; 3 Department of Sports Performance, National Institute of Fitness and Sports in Kanoya, Shiromizu-cho 1, Kagoshima, 891-2393, Japan; 4 Department of Food and Nutrition, Japan Women's University, 2-8-1 Mejirodai, Bunkyo-ku, Tokyo, 112-8681, Japan; 5 Institute of Health and Sport Sciences, University of Tsukuba, 1-1-1 Tennodai, Tsukuba, 305-8574, Japan

## Abstract

**Background:**

It has been thought that intramuscular ADP and phosphocreatine (PCr) concentrations are important regulators of mitochondorial respiration. There is a threshold work rate or metabolic rate for cellular acidosis, and the decrease in muscle PCr is accelerated with drop in pH during incremental exercise. We tested the hypothesis that increase in muscle oxygen consumption (o_2mus_) is accelerated with rapid decrease in PCr (concomitant increase in ADP) in muscles with drop in pH occurs during incremental plantar flexion exercise.

**Methods:**

Five male subjects performed a repetitive intermittent isometric plantar flexion exercise (6-s contraction/4-s relaxation). Exercise intensity was raised every 1 min by 10% maximal voluntary contraction (MVC), starting at 10% MVC until exhaustion. The measurement site was at the medial head of the gastrocnemius muscle. Changes in muscle PCr, inorganic phosphate (Pi), ADP, and pH were measured by ^31^P-magnetic resonance spectroscopy. o_2mus _was determined from the rate of decrease in oxygenated hemoglobin and/or myoglobin using near-infrared continuous wave spectroscopy under transient arterial occlusion. Electromyogram (EMG) was also recorded. Pulmonary oxygen uptake (o_2pul _) was measured by the breath-by-breath gas analysis.

**Results:**

EMG amplitude increased as exercise intensity progressed. In contrast, muscle PCr, ADP, o_2mus_, and o_2pul _did not change appreciably below 40% MVC, whereas above 40% MVC muscle PCr decreased, and ADP, o_2mus_, and o_2pul _increased as exercise intensity progressed, and above 70% MVC, changes in muscle PCr, ADP, o_2mus_, and o_2pul _accelerated with the decrease in muscle pH (~6.78). The kinetics of muscle PCr, ADP, o_2mus_, and o_2pul _were similar, and there was a close correlation between each pair of parameters (r = 0.969~0.983, p < 0.001).

**Conclusion:**

With decrease in pH muscle oxidative metabolism accelerated and changes in intramuscular PCr and ADP accelerated during incremental intermittent isometric plantar flexion exercise. These results suggest that rapid changes in muscle PCr and/or ADP with mild acidosis stimulate accelerative muscle oxidative metabolism.

## Background

Skeletal muscle respiratory control is a cardinal issue in the field of muscle energetics. Early work on isolated mitochondria identified ADP as an important stimulator of mitochondrial respiration [1]. Thereafter, it has been verified that ADP is a control signal of muscle oxidative phosphorylation in many studies [2-7]. During steady state phase of muscle contraction, muscle O_2 _consumption (o_2mus_) linearly correlates with intramuscular phosphocreatine (PCr) concentration at varying intensities under relatively stable muscle pH conditions [8-10]. It has also been demonstrated that muscle PCr and pulmonary oxygen uptake (o_2pul_) show similar kinetics during the transition from rest to steady state exercise in humans in a non-steady state condition [11-13]. In addition, Rossiter et al. [14] demonstrated that muscle PCr and slowly developing supplementary component (slow component) of o_2pul _show similar response during a high intensity constant load exercise with decreased pH condition. Therefore, it has been thought that intramuscular ADP and PCr concentrations are important regulators of skeletal muscle oxidative metabolism [1-14].

Although o_2pul _has been used as an indicator of muscle oxidative metabolism [11-14], it does not specifically indicate oxygen consumption each of the exercising muscle group(s). Near-infrared continuous wave spectroscopy (NIR_cws_) has unique capability for non-invasively evaluating of O_2 _kinetics in an objective portion of tissue with high-time resolution. NIR_cws _was first applied to the study of exercising skeletal muscle in humans in 1991 [15]. Since then, many more groups have applied this technique [16-20]. o_2mus _can be determined using NIR_cws _with transient arterial occlusion [8], and its validity was confirmed [21]. The rate of decrease in oxygenated hemoglobin and/or myoglobin (HbO_2_/MbO_2_) under conditions in which interruption of the O_2 _supply to the muscle (arterial occlusion) reflects o_2mus _[8,21,22]. Therefore, this NIR_cws _technique enables us to determine o_2mus _during exercise where metabolic condition changes diversely.

It has been reported that there is a threshold work rate or metabolic rate for cellular acidosis (pH_T_) and that, above pH_T_, the decrease in muscle PCr is accelerated during incremental exercise [23-25]. If muscle oxidative metabolism is closely related with muscle PCr even under acidotic condition, it would be predicted that acceleration in increase in o_2mus _coincided with decrease in pH. However, there is no evidence for the effect of decrease in pH on muscle oxidative metabolism during incremental exercise.

The aim of this study was to measure o_2mus_, ADP, and PCr during incremental exercise where muscle pH changed from stable to decreasing condition. We hypothesized that the increase in o_2mus_, increase in ADP and decrease in PCr occurred similar kinetics throughout incremental exercise. When exercise intensity increased above pH_T_, there is a possibility that the accelerative decrease in PCr stimulates accelerative increase in muscle oxidative metabolism during incremental exercise. To test the second hypothesis that with decrease in pH accelerative decrease in PCr could be responsible for the increase in o_2mus_, we identified the inflexion point of pH, PCr, ADP, cytosolic free energy of ATP hydrolysis (ΔG_ATP_), o_2mus_, and o_2pul _during incremental exercise. We predicted that when exercise intensity increased above the level which decrease in pH occurred, PCr, ADP, ΔG_ATP_, o_2mus_, and o_2pul _would show greater change than that obtained during stable pH condition during incremental exercise.

## Methods

### Subjects

Five male volunteers, aged between 22 and 34 years, participated in this study. All subjects were healthy, non-smokers, and free of known diseases. All subjects were fully informed of the risks involved in this study, and we obtained written informed consent from each. This study was approved by the Institutional Committee for the protection of human subjects.

### Experimental design

Each subject sat on a platform in an upright sitting position with his right leg positioned horizontally. The subjects performed the same exercise procedure five times on different occasions: once (day 1) with the 31-phosphorus-magnetic resonance spectroscopy (^31^P-MRS) measurement, twice (day 2, 3) with the respiratory gas analysis, once (day 4) with the NIR_cws _measurement for determination of o_2mus_, and once (day 5) with the EMG record. With the exception of the ^31^P-MRS measurement, the other four measurements were performed outside the MRS magnet. During these four measurements the subjects inserted a leg into a cylindrical plastic pipe of the same diameter and length as the bore of the MRS magnet. For each measurement, whether in the magnet or the plastic pipe, the leg was held in a fixed position by a cradle.

### Exercise Protocols

On occasions of the experiment, maximal voluntary contractions (MVC) was measured prior to the principal experiment, and each subject's exercise load was set based on the MVC of each. The MVC of isometric plantar flexion was measured by pushing against a foot pedal with connected force transducer. MVC was measured three times with sufficient rest (> 3 min) between each performance. The maximum value was used as the MVC. After sufficient rest in an upright sitting position, the subjects performed repetitive intermittent isometric plantar flexion exercise with the right leg in the same position. One duty cycle of contraction and relaxation consisted of a 6-s contraction and a 4-s relaxation. With the use of a visual feedback meter, the subjects were directed to perform using the prescribed force. Additionally, the experimental director continuously verified force. Exercise intensity was increased incrementally every 60 s by 10% MVC, starting from 10% MVC to an intensity at which the subject could no longer maintain the required force. A backrest was placed behind the subject during exercise. To fix position of the subject and to limit involvement of muscles other than calf muscle, the contact area of the backrest and subject's body was limited as small as possible. The height of backrest was 21 cm, and area of contact against subject's body was limited to lower back only. The subjects were instructed not to exert muscles other than the calf muscle to the best of their ability during the exercise, and they were fully familiarized with the exercise prior to the experiment.

### ^31^P-MRS

^31^P-MRS signals were obtained by an NMR spectrometer (Otsuka Electronics Co. Ltd.) with a 2.0-T superconducting 26-cm bore magnet. A double tuned (^1^H and ^31^P), 3.0-cm diameter radio frequency surface coil tuned to 34.58 MHz with 60-μs pulse width was used for the phosphorus signal. Pulse repetition time was 2 s. Five pulses were averaged to obtain a free induction decay (FID). Therefore, a spectrum was obtained every 10 s. Twelve spectra were averaged during the pre-exercise resting period, and three spectra were averaged during exercise. The surface coil was placed on the medial head of the gastrocnemius muscle (m.MG), and the coil and leg were held in a fixed position in the magnet by a cradle. All ^31^P-MRS spectra were fitted to a Lorentzian line shape using the least-squares method. The relative area and frequency of the individual peaks were determined (Otsuka Electronics software) to calculate the areas of PCr, inorganic phosphate (Pi), and β-ATP peaks. The PCr and Pi intensities were normalized using the sum of PCr and Pi to avoid influence from possible changes in the sensitivity of ^31^P-MRS signals. Saturation correction was performed using saturation factors of PCr, Pi, and β-ATP peaks, which were calculated by comparing the data from the 2-s and fully relaxed spectra. The saturation factors of PCr, Pi, and β-ATP peaks in this study were 1.330, 1.081, and 1.184, respectively. The intracellular pH was calculated from the median chemical shift between the P_i _and PCr peaks [26]. Changes in muscle PCr are expressed as a percentage of the pre-exercise resting value.

To convert peak areas to concentrations, the β-ATP peak was assumed to represent total ATP and was set at 8.2 mM [27-29]. [PCr] and [Pi] could then be estimated as the product of the areas to ATP (as PCr to β-ATP and Pi to β-ATP) and 8.2 mM. Total creatine (TCr) was assumed to be equal to the sum of PCr and Pi ([TCr] = [PCr] + [Pi]), and TCr was assumed to be constant throughout the experiment [10]. ADP was calculated with the assumption that equilibrium of the Cr kinase (CK) reaction [23,30,31]:

[ADP] = {0.74 [ATP]([TCr] - [PCr])} / {(1.66 × 10^9^)(10^-pH_obs_) [PCr]}     (*1*)

The constant 0.74 is the estimated monovalent ion activity coefficient [31] that corrects for the fact that pH_obs _is an activity, subscript obs indicates observed factors, and 1.66 × 10^9 ^is the equilibrium constant for CK. Free magnesium was assumed to be 1 mM and unchanging throughout the experiment [32]. Cytosolic free energy of ATP hydrolysis (ΔG_ATP_) was also calculated [23,30,31]:

ΔG_ATP _= ΔG_O _+ RT ln ([ADP] [Pi] / [ATP]) + RT ln [10^-(pH_obs_-7)]     (*2*)

ΔG_O _is Gibb's free energy, R is gas constant, and T is absolute temperature. ΔG_O _is taken to be -32 kJ/mol at pH7.0[31], RT at 37°C is 2.58.

### NIR spectroscopy

NIR signals were obtained by NIR_cws _(HEO-200, OMRON Co. Ltd.). The NIR_cws _probe contained a light source and an optical detector with a distance of 3.0 cm between the light source and detector to provide sensory input for the unit. A pair of two-wavelength light emitting diodes, with wavelengths of 760 and 840 nm, was used as the light source. A silicon photodiode was used as the photodetector. The NIR_cws _probe was placed on the m.MG, and the probe and leg were held in a fixed position by a cradle in a plastic pipe that mimicked the bore of the MRS magnet. Changes in HbO_2 _and/or MbO_2_, deoxygenated Hb and/or Mb, and total hemoglobin and/or myoglobin (THb/TMb) were calculated by the least squares method using data from the changes in the absorbance of these different wavelengths of light. The sampling time of the data was 0.1 s.

o_2mus _was measured using NIR_cws _with the transient arterial occlusion technique described previously in detail [8,21,22]. o_2mus _was determined by the rate of decrease in HbO_2_/MbO_2 _during arterial occlusion. Since the changes in HbO_2_/MbO_2 _measured by NIR_cws _show a dynamic balance between O_2 _supply and O_2 _consumption, the rate of decrease in HbO_2_/MbO_2 _during arterial occlusion reflects the o_2mus _[8,21,22]. Arterial occlusion was performed for 1 min during rest, and for 6 s once every 30 s during isometric contraction. Timing for arterial occlusion during exercise took place at the third and the sixth contraction of each intensity i.e. at 20–26 s and 50–56 s of each minute. The o_2mus _was expressed as a value relative to that obtained at rest (fold of resting).

### Respiratory gas analysis

o_2pul _was measured during the pre-exercise resting period and throughout the exercise period by the breath-by-breath gas analysis method using an Aeromonitor AE-280 (Minato Medical Science Co. Ltd.) [33]. This system consists of a microcomputer, a hot-wire flow-sensor, and oxygen and carbon dioxide analyzers (zirconium element-based oxygen analyzer and infra-red carbondioxide analyzer). Prior to the experiments, the flow-sensor and gas analyzers were calibrated with a known volume of room air at several mean flow rates and gas mixtures of known concentration, respectively. To improve the signal-to-noise ratio of o_2pul_, each subject performed the exercise session for o_2pul _measurement twice on different days, and the dual measurement data were subsequently averaged.

### Surface electromyograms

Surface electromyography (EMGs) were obtained from the m.MG, lateral head of the gastrocnemius muscle (m.LG), and soleus muscle (m.SOL) using a bipolar, silver-silver chloride electrode (10 mm diameter sample area) with a fixed inter-electrode spacing of 30 mm (Nihon Koden Co., Japan) during incremental plantar flexion exercise. The EMG signal was sampled at a rate of 2000 Hz using available software (BIOPAC Systems, Inc., USA) and stored on computer disk for later analysis. The root mean square of the EMG signal (rmsEMG) was calculated. Prior to the principal experiment the subjects performed MVC, and the rmsEMG was normalized as 100% at MVC.

### Data analysis

**Figure 1:**
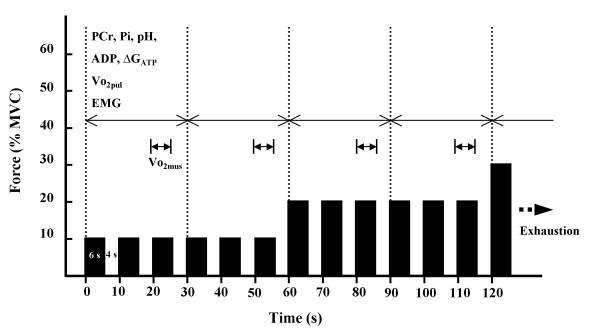
**Procedure for data analysis**. Each parameter was analyzed every 30 s. Muscle phosphocreatine (PCr), inorganic phosphate (Pi), pH, estimated ADP and free energy of ATP hydrolysis (ΔG_ATP_), pulmonary oxygen uptake (o_2pul_), and electromyogram (EMG) were averaged over 30 s. The data for muscle oxygen consumption (o_2mus_) were obtained during the third (20–26 s) and sixth (50–56 s) contractions at each intensity. The o_2mus _value of the third contraction was used to represent the first 30 s of each minute, whereas the o_2mus _value of the sixth contraction was used to represent the last 30 s of each minute.  Division of data analysis (30s).  o_2mus _measurement (6 s; once per three contraction phases).

Analysis of each parameter was performed every 30 s as the procedure is shown in figure 1. Except for o_2mus_, all data were averaged over 30 s. The data for o_2mus _were obtained at the third (20–26 s) and sixth (50–56 s) contractions of each intensity. The reason o_2mus _was measured only once during three contraction phases was to avoid the limitations to exercise performance caused by interrupting the blood flow. The value of the third contraction was used to represent the first 30 s of each minute, and the value of the sixth contraction was used to represent the last 30 s of each minute. All averaged data were shown from pre-exercise rest to the first 30 s at 80% MVC exercise at which every subject was able to perform. The logarithms of the individual metabolic parameters (pH, PCr, ADP, ΔG_ATP_, o_2mus _o_2pul_) were plotted against exercise intensity in order to determine a break point of metabolic change based on the method of determining lactate threshold [34]. These plots were best fit by a piecewise linear regression model with a breakpoint.

### Confirmation of reproducibility

Since the subjects performed the same exercise procedure five times, we were able to obtain five sets of performance data. The maximal exercise intensity the subjects were able to perform during the exercise protocol was 80–90% MVC (450–510 s). The maximal intensity at which each subject was able to perform was the same throughout five exercise sessions. The coefficient of variation for exercise duration was 0.91%. Regarding time course change and peak value, o_2pul _did not differ significantly during the two measurements. There was a significant correlation between each time measurement for individual pulmonary o_2_(r = 0.981~0.993, p < 0.001).

### Statistical analyses

Data are expressed as means ± SD. The data were compared to determine significant changes in the values of each parameter every 30 s compared with the values obtained during the first 30 s of exercise (the first 30 s at 10% MVC), and the 30 s of exercise immediately before. One-way analysis of variance (ANOVA) for repeated measures was used to determine the significance of time course changes in each parameter, and Fisher's PLSD post hoc comparisons were used to determine the significance of differences of each parameter every 30 s. A linear regression analysis was used to examine the relationship between each parameter. P < 0.05 was defined as statistically significant.

## Results

**Figure 2:**
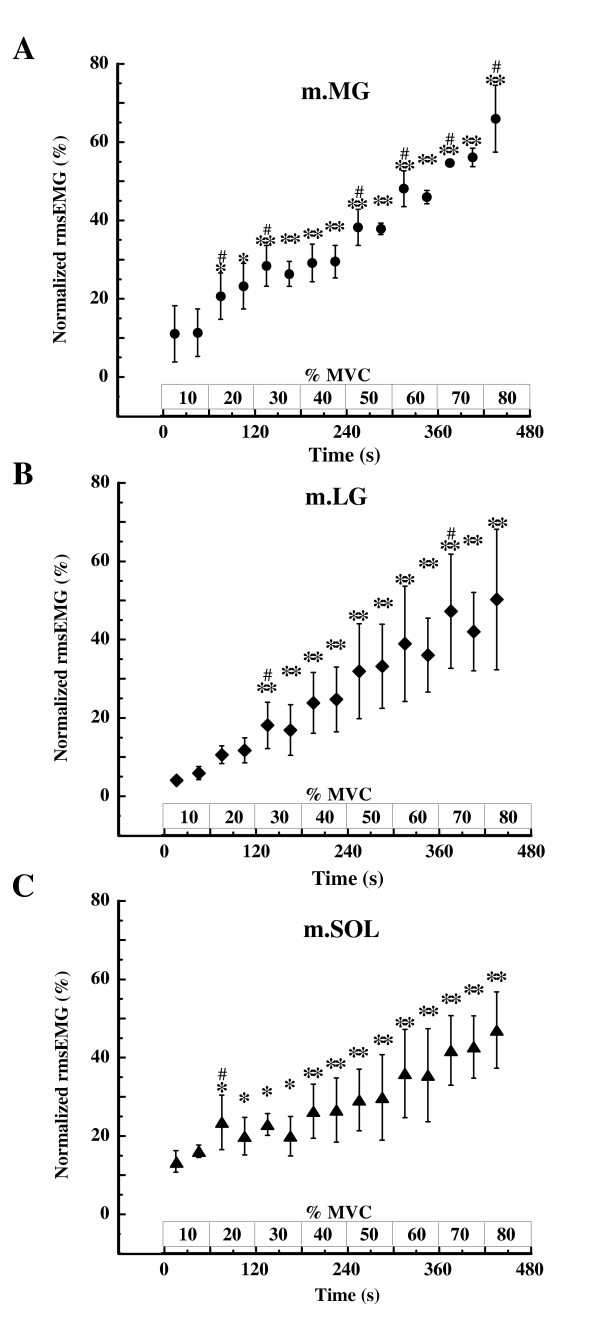
**Changes in root mean square of EMG (rmsEMG) during incremental intermittent isometric plantar flexion exercise**. Changes in rmsEMG at (A) the medial head of the gastrocnemius muscle (m.MG), (B) the lateral head of gastrocnemius muscle (m.LG), and (C) the soleus muscle (m.SOL) during incremental intermittent isometric plantar flexion exercise. Data are represented as relative values obtained during maximal voluntary contraction (MVC) as 100%. Values shown are means ± SD of 5 subjects. * p < 0.05, ** p < 0.01 vs. the value during the first 30 s at 10% MVC (first 30 s of exercise). #p < 0.05 vs. the value obtained during the 30 s of exercise immediately before.

Fig. 2 shows the time course changes in normalized rmsEMG of m.MG, m.LG, and m.SOL. The rmsEMG in those muscles increased similarly with increasing exercise intensity. The rmsEMG of m.MG for each of the first 30 s at 20%, 30%, 50%, 60%, 70%, and 80% MVC differed significantly from that during the 30 s of exercise immediately before (i.e., prior intensity) (p < 0.05). Throughout the exercise, the change in rmsEMG of m.MG was largest in the three muscle groups.

**Figure 3:**
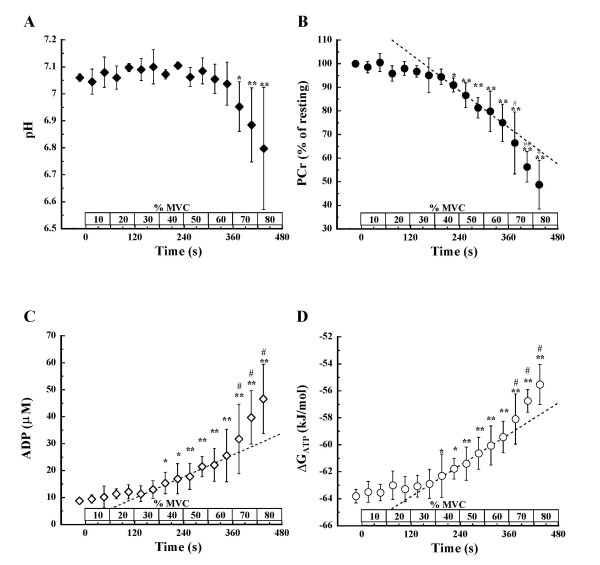
**Changes in muscle pH, PCr, ADP, and ΔG_ATP _during incremental intermittent isometric plantar flexion exercise**. Changes in (A) pH, (B) PCr, (C) ADP, and (D) ΔG_ATP _during incremental intermittent isometric plantar flexion exercise. A dotted line in each panel B, C, and D represents a linear regression line which is drawn to obtain the highest correlation coefficient above 40% MVC, at which significant difference was observed when compared with the value obtained during the first 30 s at 10% MVC. Values shown are means ± SD of 5 subjects. * p < 0.05, **p < 0.01 vs. the value during the first 30 s at 10% MVC (first 30 s of exercise). #p < 0.05 vs. the value obtained during the 30 s of exercise immediately before.

Fig. 3A shows the time course of changes in intramuscular pH. We found that pH was relatively constant, from resting values (7.06 ± 0.01) until 60% MVC (7.04 ± 0.08), but it decreased significantly (p < 0.05) at 70% MVC and with exercise progression, being 6.78 ± 0.22 at the end of exercise.

Fig. 3B shows the time course changes in intramuscular PCr. We found that there were significant differences after the last 30 s at 40% MVC when compared with the value obtained during the first 30 s at 10% MVC (p < 0.05), and that PCr decreased with progression of exercise. Above 70% MVC, the values were significantly different when compared with those obtained during the 30 s of exercise immediately before. A linear regression line was drawn to obtain the highest correlation coefficient above the last 30 s of 40% MVC, at which significant difference was observed when compared with the value obtained during the first 30 s at 10% MVC. The PCr deviated downward from the regression line above 70% MVC.

Fig. 3C shows the time course changes in estimated ADP. We found that ADP slightly increased from rest (8.8 ± 0.9 μM) until the last 30 s of 30% MVC (13.0 ± 3.2 μM), whereas above 40% MVC, these values differed significantly from those obtained during the first 30 s at 10% MVC (p < 0.05). Thereafter, ADP increased with progression of exercise, being significantly different above 70% MVC compared with the value obtained during the 30 s of exercise immediately before. At the end of exercise, ADP was 46.6 ± 12.8 μM. A linear regression line was drawn to obtain the highest correlation coefficient above the first 30 s of 40% MVC, at which significant difference was observed when compared with the value obtained during the first 30 s at 10% MVC. The ADP deviated upward from the regression line above 70% MVC.

Fig. 3D shows the time course changes in estimated ΔG_ATP_. We found that ΔG_ATP _changed only slightly from rest (-63.8 ± 0.5 kJ/mol) until the last 30 s of 30% MVC (-62.9 ± 1.1 kJ/mol), whereas above 40% MVC, these values differed significantly from those obtained during the first 30 s at 10% MVC (p < 0.05). Thereafter, ΔG_ATP_increased with progression of exercise, being significantly different above 70% MVC compared with the value obtained during the 30 s of exercise immediately before. At the end of exercise, ΔG_ATP _was -55.1 ± 1.9 kJ/mol. A linear regression line was drawn to obtain the highest correlation coefficient above the first 30 s of 40% MVC, at which significant difference was observed when compared with the value obtained during the first 30 s at 10% MVC. The ΔG_ATP _deviated upward from the regression line above 70% MVC.

**Figure 4:**
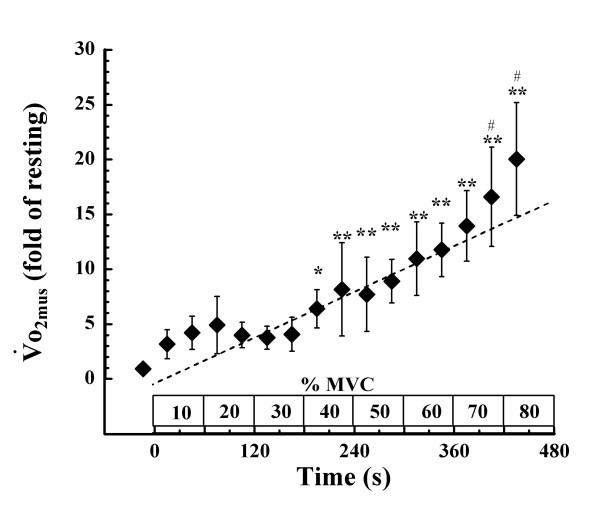
**Changes in ****o_2mus _during incremental intermittent isometric plantar flexion exercise**. A dotted line represents a linear regression line which is drawn to obtain the highest correlation coefficient above the first 30 s of 40% MVC at which significant difference was observed when compared with the value obtained during the first 30 s at 10% MVC. The o_2mus _is expressed as a value relative to that obtained at rest (fold of resting). Values shown are means ± SD of 5 subjects. *p < 0.05, **p < 0.01 vs. the value during the first 30 s at 10% MVC (first 30 s of exercise) # p < 0.05 vs. the value obtained during the 30 s of exercise immediately before.

Fig. 4 shows the time course changes in o_2mus_. o_2mus _also showed slight changes during exercise below 40% MVC. Above 40% MVC, however, there were significant differences when compared with the value obtained during the first 30 s at 10% MVC. o_2mus _subsequently increased with progression of exercise, and the values obtained during the last 30 s at 70% MVC and the first 30 s at 80% MVC differed significantly from the value during the 30 s of exercise immediately before. The peak value of o_2mus _was 21.3 ± 5.2 fold higher than its resting value. A linear regression line was drawn to obtain the highest correlation coefficient above the first 30 s of 40% MVC, at which significant difference was observed when compared with the value obtained during the first 30 s at 10% MVC. The o_2mus _deviated upward from the regression line above 70% MVC.

**Figure 5:**
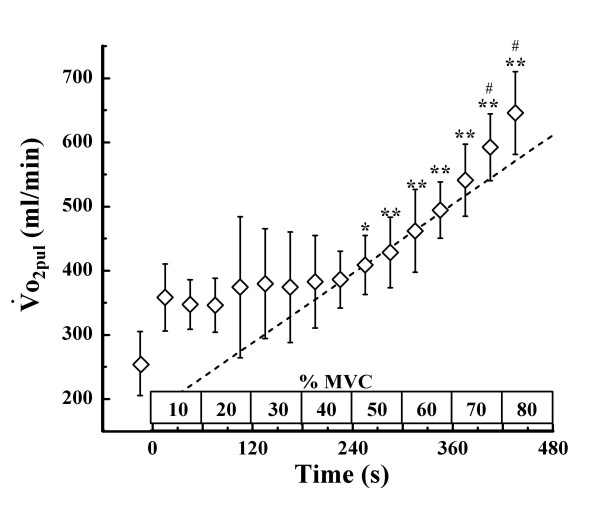
**Changes in ****o_2mus _during incremental intermittent isometric plantar flexion exercise**. A dotted line represents a linear regression line which is drawn to obtain the highest correlation coefficient above the first 30 s of 50% MVC at which significant difference was observed when compared with the value obtained during the first 30 s at 10% MVC Values shown are means ± SD of 5 subjects. * p < 0.05, **p < 0.01 vs. the value during the first 30 s at 10%MVC(first 30 s of exercise), # p < 0.05 vs. the value obtained during the 30 s of exercise immediately before.

Fig. 5 shows the time course changes in o_2pul_. We found that o_2pul _changed only slightly, and there was little difference relative to exercise intensity up to 40% MVC. When the exercise intensity was raised above 50% MVC, the value of o_2pul _differed significantly from that obtained during the first 30 s at 10% MVC. Thereafter, o_2pul _increased with progression of exercise, and the values obtained during the last 30 s at 70% MVC and the first 30 s at 80% MVC were significantly different from the value obtained during the 30 s of exercise immediately before. The peak value of of o_2pul _was 684.8 ± 64.8 ml/min, which different from the resting value of o__2_pul _(Δo_2pul_) by 364.8 ± 74.3 ml/min. A linear regression line was drawn to obtain the highest correlation coefficient above the first 30 s of 50% MVC, at which significant difference was observed when compared with the value obtained during the first 30 s at 10% MVC. The o_2pul _deviated upward from the regression line above 70% MVC.

**Figure 6:**
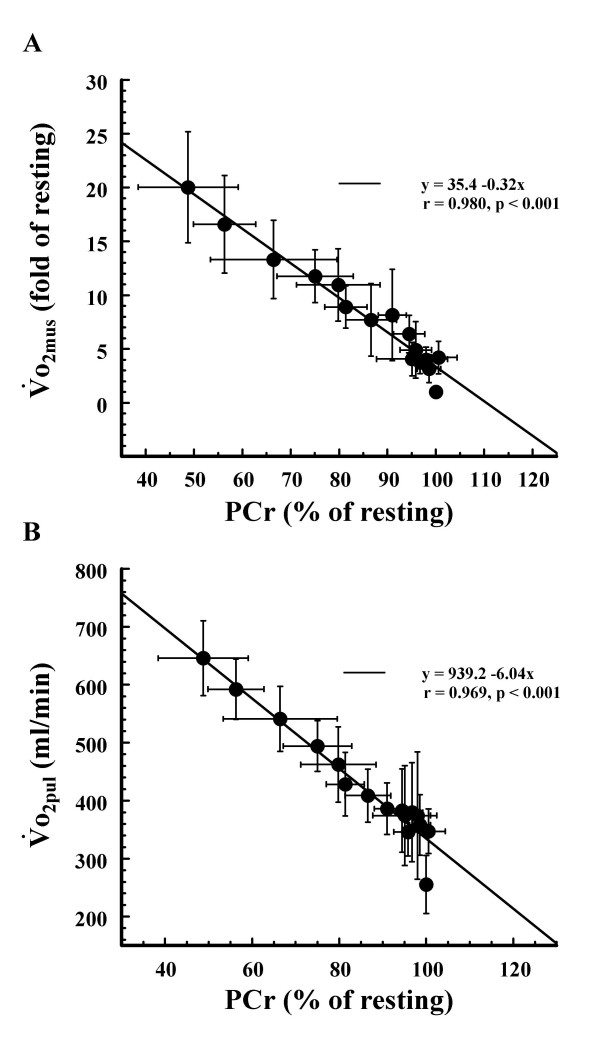
**Relationships between muscle PCr and ****o_2mus_, muscle PCr and ****o_2pul_**. Relationships between (A) averaged muscle PCr and o_2mus _and (B) averaged muscle PCr and o_2pul_. Values shown are means ± SD of 5 subjects.

When we examind the relationship between the averaged muscle PCr and the averaged o_2mus_, we observed a significant inverse correlation between the two (r = 0.980, p < 0.001) (Fig 6A). There was also a significant inverse correlation between averaged muscle PCr and averaged o_2pul _(r = 0.969, p < 0.001) (Fig 6B).

**Figure 7:**
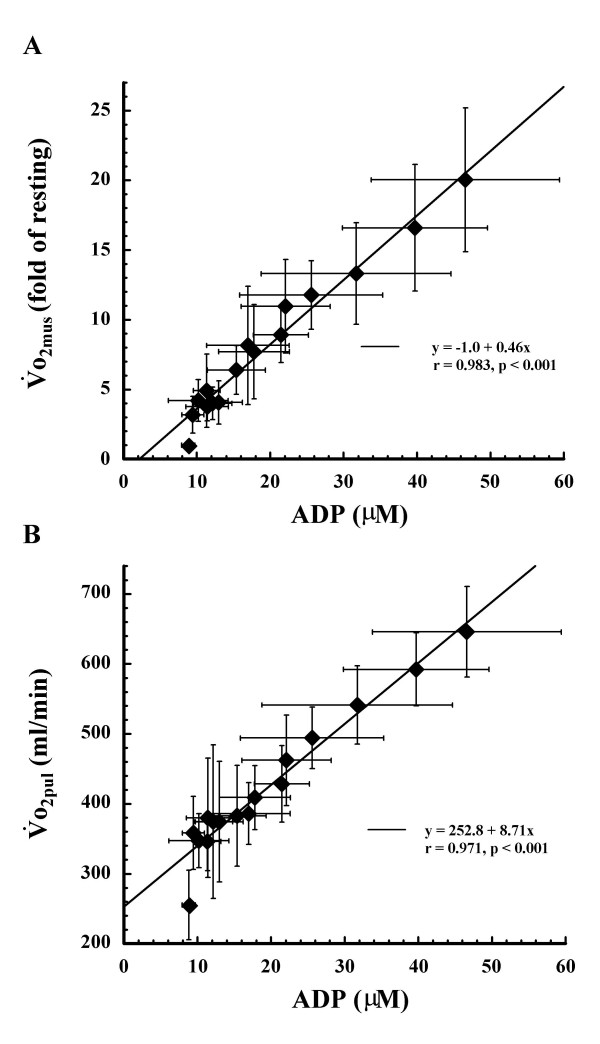
**Relationships between muscle ADP and ****o_2mus_, muscle ADP and ****o_2pul_**. Relationships between (A) averaged muscle ADP and o_2mus _and (B) averaged muscle ADP and o_2pul_. Values shown are means ± SD of 5 subjects.

We also determined the relationship between the averaged ADP and the averaged o_2mus _(Fig. 7A) and between the averaged ADP and the averaged o_2pul _(Fig. 7B). There was a significant correlation betwene ADP and o_2mus _(r = 0.983, p < 0.001) and between ADP and o_2pul _(r = 0.971, p < 0.001). Individual correlation between ADP and o_2mus_(r = 0.916~0.963, p < 0.001) and between ADP and o_2pul _(r = 0.902~0.974, p < 0.001) were seen in all subjects (Figures not shown). Additionally, there was a significant positive correlation between averaged o_2mus _and averaged o_2pul _(r = 0.975, p < 0.001) (Figure not shown).

The logarithms of individual metabolic parameters (pH, PCr, ADP, ΔG_ATP_, o_2mus_, o_2pul_) were best fit by the piecewise regression model with an inflexion point ranging from 60 to 70% MVC, and individual break points for all metabolic parameters were the same intensity in all subject. There were significant intra-individual correlations between each pair of metabolic parameters (r = 0.971~0.988, p < 0.001). 0.001)

## Discussion

The main finding of this study was that increase in o_2mus _accelerated coincidentally with drop in muscle pH over 70% MVC during incremental intermittent isometric contraction. Changes in muscle PCr ADP, ΔG_ATP_, and o_2pul _also accelerated simultaneously with drop in pH. In addition, the kinetics of each metabolic parameter was similar, and there were significant correlations between each pair of parameters (r = 0.969~0.983, p < 0.001).

It has been thought that intramuscular ADP and PCr concentration are important regulators of mitochondrial respiration [1-14,35,36]. However, there is no evidence that examined relationship between muscle oxidative metabolism and muscle PCr or ADP during incremental exercise where muscle pH changed from stable to decreasing condition. According to the PCr shuttle hypothesis [37] and other biochemical hypotheses [38], control of respiration is exerted linearly at the mitochondria by the declining PCr and concomitant rise in cytosolic Cr. These hypotheses [37,38] are based on observations of linear changes in muscle respiration relative to increasing contraction intensity under relatively stable pH conditions. The greater rate of breakdown of PCr under acidotic conditions [23-25], if still tightly coupled to oxidative phosphorylation, would predict that o_2mus _increases nonlinearly with increasing contraction intensity. In this study, the increase in o_2mus _is actually accelerated with rapid decrease in PCr during a conspicuous drop in pH, to ~ 6.78. Additionally, the accelerated increase in o_2mus _coincided with abrupt increase in ADP. Consequently, our results indicated that muscle oxidative metabolism is closely related with muscle PCr and ADP even under mild acidotic conditions. Therefore, it is suggested that rapid changes in muscle PCr and/or ADP, coincided with drop in pH, are factor(s) that accelerate muscle oxidative metabolism during incremental intermittent isometric contraction.

The accelerated changes in PCr, ADP, o_2mus_, o_2pul_, and the calculated ΔG_ATP _above 70% MVC coincided with the decrease in pH, indicating that metabolic demand changes nonlinearly with increasing exercise intensity (Fig. 3, Fig. 4, Fig. 5). In contrast, others have shown that, although above pH_T _muscle PCr rapidly decreases, ΔG_ATP _increases linearly with increasing intensity throughout dynamic plantar flexion exercise [23]. o_2pul _rises linearly with increasing work rate during bicycle exercise [39,40], and ΔG_ATP _shows a linear increase with increasing work rate during dynamic plantar flexion exercise [23] consistent with pulmonary o_2 _[39,40]. One possible explanation for the different results between the earlier studies [23,39,40] and ours is the difference of load setting. Previous dynamic exercise studies were incremented by prescribed work rate, until either the frequency of contraction/s or the full range of motion could no longer be sustained [23] or until maximal o_2pul _was attained by increments of 15–30W/min ramp loaded bicycle exercise [39,40]. In contrast, we loaded using % MVC and reached 80–90% MVC at the end of exercise. Although % MVC was not expressed in those previous studies, it is possible that the peak intensity attained in our study was higher in those previously reported [23,39,40]. It is therefore conceivable that a larger amount of type II fibers were recruited in our study during exercise above the intensity where drop of muscle pH occurred. Since the energy cost of type II fibers is larger than that of type I fibers [41,42], an increase in type II fiber recruitment may produce greater changes in muscle PCr, ADP, o_2mus_, o_2pul _and ΔG_ATP _above the intensity during which a decrease in pH occurs.

Another explanation for the different results between the earlier study [23] and ours (i.e. linear vs. nonlinear increase in ΔG_ATP_) may be the difference in types of muscle contraction (i.e. concentric vs. intermittent isometric contraction). A nonlinear relationship between heat production, an indicator of ATP turnover rate, and force production during voluntary isometric contractions has been reported, although EMG activity continued to increase linearly with force production [43]. In addition, it is impossible to determine mechanical work for this type of static contraction. Therefore, voluntary isometric contraction does not necessarily show linear relationships between energy demand and exercise intensity or muscle electrical activity.

One might criticize that despite the increasing exercise intensity in the initial phases, up to 30–40% MVC, there were only small changes in energy metabolism. At the onset of exercise, o_2pul _showed a steep increase, which remained stable until 40% MVC. o_2pul _and heart rate often exceed their steady state levels at the onset of exercise (phase I) during very low work rates [44]. This abrupt increase in o_2pul _is due to the rapid elevation of cardiac output that drives mixed venous blood through the lungs [44]. It is possible, therefore, that phase I o_2pul _exceeded the oxygen demand from the initial phase of exercise in this study. PCr, ADP, ΔG_ATP_, o_2mus_, and o_2pul _also changed only slightly during exercise below 30–40% MVC. We found however, that rmsEMG of m.MG, the same site as ^31^P-MRS and NIR_cws _measurements, increased with increasing exercise intensity, and that rmsEMG of m.LG and m.SOL changed similarly with m.MG (Fig. 2). These results indicate that, although energy consumption changed slightly below 30–40% MVC, muscle electrical activity changed significantly with increased exercise intensity. It has been demonstrated that heat production increased only moderately with increasing contraction intensity during isometric contraction at low intensities, though EMG increased relative to contraction intensity [43]. Therefore, it appears that little metabolic change during exercise at low intensities is a characteristic of isometric contraction.

One limitation of our study is that the bore diameter of the ^31^P-MRS magnet used in this study was small (26cm), and it only permitted us to perform intermittent isometric plantar flexion. Isometric contraction is sensitive to occlude blood flow [45]. Therefore, one concern is that limited blood flow affected the results of this study. However, as far as we observe EMGs, the subjects fully relaxed between contractions even at highest intensities. In addition, metabolic parameters (pH, PCr, ΔG_ATP_) of this study reached at the end of exercise were approximately same levels as reported data which performed incremental dynamic plantar flexion exercise [23]. The result obtained in this study could be comparable to the previous study that used dynamic exercise [23].

Although EMGs of plantar flexor muscles increased with increasing exercise intensity it is impossible to entirely eliminate the possibility that increases in o_2pul _could include o_2mus _from other muscles besides the plantar flexors. These should include muscles that maintain posture during exercise especially at high intensity. However, we observed a linear relationship between calf o_2mus _and o_2pul _(r = 0.975, p < 0.001). Moreover, the o_2pul _kinetics was similar to muscle ADP and PCr which are thought to be important regulators of muscle oxidative metabolism. Therefore, we believe that the increase in o_2pul _primarily derives from the increase in o_2mus _in the active calf muscle.

## Conclusion

o_2mus _changed similarly with PCr and ADP throughout incremental intermittent isometric plantar flexion exercise. The increase in o_2mus _accelerated under mild acidosis during exercise at high intensity. The point of acceleration coincided with rapid changes in muscle PCr and ADP. The results of this study suggest that rapid decrease in PCr (concomitant accelerative increase in ADP) under mild acidotic condition stimulates accelerative muscle oxidative metabolism during incremental intermittent isometric exercise at high intensity.

## Authors' contributions

Toshiyuki Homma conceived the experimental design, carried out the experiment, and drafted the manuscript. Takafumi Hamaoka, M.D., Ph.D, participated in the design and coordination of the study, and directed throughout the study. Takayuki Sako, Ph.D., Motohide Murakami, M.D., Ph.D., Kazuki Esaki and Ryotaro Kime, Ph.D, participated in the study design and carried out the experiment. Toshihito Katsumura, MD., Ph.D, participated in the design and coordination of the study. All authors read and approved the final manuscript.
